# The Relationship of Initial Transferrin Saturation to Cardiovascular Parameters and Outcomes in Patients Initiating Dialysis

**DOI:** 10.1371/journal.pone.0087231

**Published:** 2014-02-05

**Authors:** Hyang Mo Koo, Chan Ho Kim, Fa Mee Doh, Mi Jung Lee, Eun Jin Kim, Jae Hyun Han, Ji Suk Han, Hyung Jung Oh, Jung Tak Park, Seung Hyeok Han, Tae-Hyun Yoo, Shin-Wook Kang

**Affiliations:** 1 Department of Internal Medicine, Yonsei University College of Medicine, Seoul, Korea; 2 Brain Korea 21 PLUS Project for Medical Science, Yonsei University, Seoul, Korea; The Pennsylvania State University Hershey Medical Center, United States of America

## Abstract

**Background:**

The prognostic importance of anemia for cardiovascular (CV) events and mortality has been extensively investigated. However, little is known about the impact of transferrin saturation (TSAT), a marker reflecting the availability of iron for erythropoiesis, on clinical outcome in dialysis patients.

**Methods:**

A total of 879 anemic incident dialysis patients were recruited from the Clinical Research Center for End-Stage Renal Disease in Korea and were divided into 3 groups according to baseline TSAT of ≤20%, 20–40%, and >40%.

**Results:**

There were no differences in hemoglobin levels and the proportion of patients on erythropoiesis-stimulating agents or iron supplements among the 3 groups. During a mean follow-up duration of 19.3 months, 51 (5.8%) patients died. CV composite (11.71 vs. 5.55 events/100 patient-years, P = 0.001) and all-cause mortality rates (5.38 vs. 2.31 events/100 patient-years, P = 0.016) were significantly higher in patients with TSAT ≤20% compared to those with TSAT 20–40% (reference group). Cox regression analysis revealed that patients with TSAT ≤20% had 1.62- and 2.19-fold higher risks for CV composite outcome (P = 0.046) and all-cause mortality (P = 0.030). Moreover, TSAT ≤20% was significantly associated with left ventricular hypertrophy [odds ratio (OR)  = 1.46], high-sensitivity C-reactive protein ≥3 mg/dL (OR = 2.09), N-terminal pro B-type natriuretic peptide ≥10000 pg/mL (OR  = 2.04), and troponin-T≥0.1 ng/mL (OR  = 2.02), on logistic regression analysis.

**Conclusions:**

Low TSAT was a significant independent risk factor for adverse clinical outcome in incident dialysis patients with anemia, which may be partly attributed to cardiac dysfunction and inflammation.

## Introduction

Anemia is prevalent in patients with chronic kidney disease (CKD), and develops during the early stages of the disease and worsens as renal function declines [1], [2]. It is well known that anemia is closely linked with fatigue, exercise intolerance, and poor quality of life. In addition, anemia has been demonstrated to be an independent risk factor for left ventricular hypertrophy (LVH), congestive heart failure (CHF), and cardiovascular (CV) mortality [3]–[5]. In CKD patients, anemia is also associated with the progression of renal dysfunction [6], [7].

Anemia in CKD patients is attributed to inadequate production of erythropoietin, iron and/or folate deficiency, secondary hyperparathyroidism, chronic inflammation, and bone marrow suppression due to uremic toxins [8]. Among these factors except for erythropoietin deficiency, iron deficiency is the leading cause of anemia in patients with CKD. Therefore clinicians must determine patients' iron levels not only at the start of erythropoiesis-stimulating agent (ESA) therapy but also monitor iron levels during ESA treatment in this population [9], [10]. To evaluate iron status, serum iron concentrations, transferrin saturation (TSAT); the ratio of serum iron to total iron-binding capacity (TIBC), multiplied by 100, and serum ferritin levels are commonly used. While serum iron concentrations and TSAT reflect the amount of iron available for erythropoiesis, serum ferritin levels are the only marker of total body iron stores. Ferritin levels also are greatly influenced by nutritional and/or inflammatory status and do not correlate well with bone marrow findings in patients with various chronic diseases [11]. Considering these findings, TSAT <20% and serum ferritin concentrations <100 ng/mL are regarded as absolute iron deficiency, and TSAT <20% and serum ferritin levels >100 ng/mL as relative iron deficiency [9], [11].

Iron is an essential nutrient. It plays critical roles in binding and transporting oxygen, oxidative metabolism by serving as a component of the mitochondrial respiratory chain proteins, and the synthesis of DNA and protein [12]. Therefore, iron deficiency may result in numerous pathologies, especially in cells with high energy demands, such as cardiomyocytes [13], [14]. In addition to its association with poor cognitive function, reduced exercise performance, and decreased quality of life, previous studies have found that absolute or relative iron deficiency, regardless of the presence of anemia, is an independent predictor of adverse clinical outcomes, including a progression of CHF and mortality, in patients with CHF [15]–[18]. These findings suggest that lack of circulating available iron has a direct deleterious effect on the heart.

Since CV disease is the most common cause of morbidity and mortality in patients with end-stage renal disease (ESRD), it follows that iron deficiency may have a negative impact on the clinical outcome of ESRD patients. However, to date, this effect has never been evaluated in this population. In the present study, therefore, we investigated whether low TSAT was a significant predictor of CV mortality/composite outcome and all-cause mortality in Korean incident dialysis patients from the Clinical Research Center (CRC) for ESRD. The relationships of TSAT with echocardiographic parameters and cardiac biomarkers were also defined in these patients.

## Patients and Methods

### Ethics statement

This study was carried out in accordance with the Declaration of Helsinki and study protocol was approved by the Institutional Review Board of each participating hospital's Clinical Trial Center (CTC); Kyungpook National University Hospital CTC, Youngnam University Medical Center CTC, Dong-A University Medical Center CTC, Busan National University Hospital CTC, Inje University Pusan Paik Hospital CTC, Ulsan University Hospital CTC, Seoul National University Hospital CTC, Seoul National University Bundang Hospital CTC, Seoul National University Boramae Hospital CTC, Gachon University Gil Medical Center CTC, Yonsei University Health System CTC, National Health Insurance Corporation Ilsan Hospital CTC, Ehwa Womens University Mokdong Hospital CTC, Kwandong University College of Medicine Myongi Hospital CTC, Kangnam Severance Hospital CTC, The Catholic University of Korea Seoul St. Mary's Hospital CTC, The Catholic University of Korea Yeouido St. Mary's Hospital CTC, The Catholic University of Korea Bucheon St. Mary's Hospital CTC, The Catholic University of Korea St. Vincent's Hospital CTC, The Catholic University of Korea Uijeongbu St. Mary's Hospital CTC, Chung-Ang University Health System CTC, Chonnam National University Hospital CTC, Chungnam National University Hospital CTC, Chungbuk National University Hospital CTC, Chonbuk National University Hospital CTC, and Cheju Halla General Hospital CTC. All patients provided their written informed consent before entering the study.

### Patients

Initial recruitment for this prospective observational multicenter study included all ESRD patients who started dialysis between August 1, 2008 and December 31, 2012 at 27 centers of the CRC for ESRD in Korea. Among these patients, we excluded those who were younger than 18 years old, had a history of kidney transplantation prior to dialysis therapy, had an underlying active malignancy or acute infection, or died within 3 months of the initiation of dialysis. Patients who were not anemic; anemia was defined as Hb <13 g/dL in men and <12 g/dL in women according to World Health Organization (WHO) criteria [19], had a recent bleeding episode, or had insufficient baseline data were also excluded from the study. Ultimately, a total of 879 incident dialysis patients were included in the final analysis.

### Data Collection

Demographic and clinical data were recorded at the time of study entry, including age, gender, body mass index (BMI) calculated as weight/height^2^, primary renal disease, comorbidities, and medications. Coronary arterial disease (CAD) was defined as a history of angioplasty, coronary artery bypass grafts, myocardial infarction, or angina, while peripheral arterial disease (PAD) was defined as a history of claudication, ischemic limb loss and/or ulceration, or peripheral revascularization procedure. At the time of study entry and every 3 months thereafter, laboratory data were measured from fasting blood samples, which were drawn prior to the start of hemodialysis (HD) on the day of a midweek session in HD patients and at 2-hours after the first peritoneal dialysis (PD) exchange with 1.5% dextrose dialysate in PD patients. The estimated glomerular filtration rate (eGFR) was calculated using the four-variable Modification of Diet in Renal Disease study (MDRD) and Chronic Kidney Disease Epidemiology Collaboration study (CKD-EPI) equations [20]. In addition, a 24-hour urine collection was performed to determine residual urine volume, and 24-hour urinary protein, urea, and creatinine excretion. Nutritional status was also evaluated by the subjective global assessment (SGA) score [21]. The quality of life was assessed using a Korean version of Kidney Disease Quality of Life Short Form (KDQOL-SF, version 1.3) [22].

Echocardiography was performed on a non-dialysis day in HD patients and in the morning with an empty abdomen in PD patients, close to the time of discharge, based on the imaging protocol recommended by the American Society of Echocardiography. Left atrial dimension (LAD) was assessed at the end of the ventricular systole at the level of the aortic valve, according to the leading-edge-to-leading-edge convention. Left ventricular (LV) mass was determined using the method described by Devereux et al. [23], and the LV mass index (LVMI) was calculated by dividing LV mass by body surface area. LV hypertrophy (LVH) was defined as a LVMI >131 g/m^2^ for men and >100 g/m^2^ for women [24]. LV systolic function was estimated by the LV ejection fraction (LVEF) using a modified biplane Simpson's method from the apical two-chamber and four-chamber views. Inter-ventricular septal thickness (IST), left ventricular posterior wall thickness (LVPWT), and left ventricular dimensions (LVEDD, LVESD) were also measured at the end of both the diastolic and systolic phases. Multiple reproducibility, inter-reader reliability, intra-reader reliability, and reader drift analyses were performed at a core echocardiography laboratory (Kyungpook National University, Daegu, Korea) on a random sample of 3% of the entire cohort each year. The intra-class correlation coefficients for the echocardiographic measures were 0.773 for LAD, 0.745 for LVMI, and 0.842 for LVEF.

### Outcome measures

For the current study, all mortality and hospitalization event records were retrieved from the CRC for ESRD database and carefully reviewed. The primary endpoints were CV mortality and CV composite outcome (death and hospitalization), and the secondary endpoint was all-cause mortality. CV event was considered death or hospitalization from myocardial infarction/ischemia, congestive heart failure, pulmonary edema, or cerebrovascular disorder.

### Statistical analysis

Statistical analysis was performed using SPSS for Windows, version 18.0 (SPSS Inc., Chicago, IL, USA). Data are expressed as mean ± standard deviation or median (interquartile range) for continuous variables, and as a number (percentage) for categorical variables. Normality of distribution was assessed by the Shapiro-Wilk test. Patients were categorized into 3 groups according to TSAT concentrations; ≤20%, 20–40%, and >40%. Patient demographics, clinical characteristics, and laboratory findings were compared among the three groups using ANOVA or Kruskal-Wallis test for continuous variables and the chi-square test for categorical variables. Cumulative survival curves for CV mortality, CV composite outcome, and all-cause mortality were created by the Kaplan-Meier method, and between-group survival was compared by a log-rank test. The independent prognostic power of TSAT for clinical outcomes was ascertained by multivariate Cox proportional hazards regression analysis, which included only the variables of a P-value <0.10 on the univariate analysis. Binary logistic regression analysis was conducted to determine the independent predictive value of TSAT for echocardiographic parameters (LVEF <60%, LVH, LVEDD ≥55 mm, LVESD ≥35 mm, and LAD ≥40 mm) and inflammatory and cardiac biomarkers [high-sensitivity C-reactive protein (hs-CRP) ≥3 mg/dL, N-terminal pro B-type natriuretic peptide (NT-proBNP) ≥10000 pg/mL, and cardiac troponin-T (cTnT) ≥0.1 ng/mL]. A P-value of less than 0.05 was considered statistically significant.

## Results

### Baseline characteristics

The baseline demographics and clinical characteristics are shown in Table 1. The mean age was 56.4±14.5 years, and 59.6% of patients were male. The most common cause of ESRD was diabetes (DM, 52.7%), followed by hypertension (16.5%). A total of 645 patients (73.4%) were treated with HD and 234 patients (26.6%) with PD. The mean values of hemoglobin (Hb) and TSAT were 8.6±1.4 g/dL and 28.4±16.0%, respectively, and the median levels of ferritin were 201.3 (103.5–363.4) ng/mL.

**Table 1:** Baseline demographic and clinical characteristics of the patients.

Variables	Total	TSAT ≤20%	TSAT 20–40%	TSAT >40%	P
N	879	282	431	166	
Age (years)	56.4±14.5	57.1±14.0	56.4±14.2	55.3±16.2	0.414
Sex (Male)	524 (59.6)	162 (57.4)	258(59.9)	104 (62.7)	0.550
BMI (kg/m^2^)	23.2±3.7	23.3±3.7	23.4±3.8	22.6±3.3	0.097
Systolic BP (mmHg)	142.4±24.7	142.2±24.7	142.7±25.4	141.7±22.9	0.898
Diastolic BP (mmHg)	77.6±15.2	76.9±15.0	77.8±15.3	78.1±15.1	0.666
Heart rate (beat per minute)	76.7±13.6	76.9±13.0	76.8±13.1	76.2±15.7	0.845
Dialysis modality (HD)	645 (73.4)	212 (75.2)	310 (71.9)	123 (74.1)	0.614
Follow-up duration (months)	19.3±11.8	19.8±11.6	19.3±11.7	18.6±12.3	0.562
Smoking status					0.878
Current smoker	103 (11.7)	31 (11.0)	51 (11.8)	21 (12.7)	
Ex-smoker	291 (33.1)	92 (32.6)	143 (33.2)	56 (33.7)	
Non-smoker	467 (53.1)	155 (55.0)	225 (52.2)	87 (52.4)	
Unknown	18 (2.0)	4 (1.4)	12 (2.8)	2 (1.2)	
Primary cause of end-stage renal disease					0.151
Diabetes	463 (52.7)	162 (57.4)	228 (52.9)	73 (44.0)	
HTN/Large vessel disease	145 (16.5)	43 (15.2)	75 (17.4)	27 (16.3)	
Glomerulonephritis	147 (16.7)	38 (13.5)	67 (15.5)	42 (25.3)	
Interstitial nephritis	9 (1.0)	3 (1.1)	5 (1.2)	1 (0.6)	
Hereditary/Congenital disease	15 (1.7)	7 (2.5)	6 (1.4)	2 (1.2)	
Others	43 (4.9)	11 (3.9)	24 (5.6)	8 (4.8)	
Unknown	57 (6.5)	18 (6.4)	26 (6.0)	13 (7.8)	
Comorbid disease					
Chronic lung disease	72 (8.2)	20 (7.1)	32 (7.4)	20 (12.0)	0.130
CAD	127 (14.4)	48 (17.0)	57 (13.2)	22 (13.3)	0.329
PAD	65 (7.4)	22 (7.8)	36 (8.4)	7 (4.2)	0.213
CVA	92 (10.5)	29 (10.3)	47 (10.9)	16 (9.6)	0.896
CHF	127 (14.4)	42 (14.9)	65 (15.1)	20 (12.0)	0.619
Arrhythmia	25 (2.8)	6 (2.1)	17 (3.9)	2 (1.2)	0.153
Diabetes	486 (55.3)	167 (59.2)	239 (55.5)	80 (48.2)	0.076
Connective tissue disease	85 (9.7)	25 (8.9)	43 (10.0)	17 (10.2)	0.853
Liver disease	81 (9.2)	17 (6.0)	39 (9.0)	25 (15.1)	0.006
CVD*	298 (33.9)	102 (36.2)	141 (32.7)	55 (33.1)	0.618
Cardiac disease†	224 (25.5)	78 (27.7)	107 (24.8)	39 (23.5)	0.563
Modified CCI	5.1±2.6	5.1±2.5	5.1±2.6	4.9±2.6	0.665
SGA >1	278 (31.6)	84 (29.8)	144 (33.4)	50 (30.1)	0.535
KDQOL-SF score	60.1±14.9	60.1±14.8	60.5±15.2	59.0±14.3	0.696
Medications					
RAS blockers	528 (60.1)	167 (59.2)	259 (60.1)	102 (61.4)	0.898
Diuretics	461 (52.4)	150 (53.2)	225 (52.2)	86 (51.8)	0.951
Beta blocker	477 (54.3)	170 (60.3)	229 (53.1)	78 (47.0)	0.019
CCB	549 (62.5)	183 (64.9)	263 (61.0)	103 (62.0)	0.576
Nitrate	34 (3.9)	10 (3.5)	13 (3.0)	11 (6.6)	0.116
Aspirin	214 (24.3)	81 (28.7)	98 (22.7)	35 (21.1)	0.106
Clopidogrel	73 (8.3)	31 (11.0)	30 (7.0)	12 (7.2)	0.139
Vitamin D	124 (14.1)	39 (13.8)	70 (16.2)	25 (15.1)	0.680
Ca based P-binders	472 (53.7)	164 (58.2)	224 (52.0)	84 (50.6)	0.182
ESA	282 (32.1)	83 (29.4)	148 (34.3)	51 (30.7)	0.358
Iron agent	319 (36.3)	95 (33.7)	160 (37.1)	64 (38.6)	0.516

Numbers in the parentheses are percentages.

*composite of CAD, PAD, CVA, CHF, and arrhythmia.

† composite of CAD, CHF, and arrhythmia.

*Abbreviations*: BMI, body mass index; BP, blood pressure; HD, hemodialysis; HTN, hypertension; CAD, coronary artery disease; PAD, peripheral artery disease; CVA, cerebrovascular attack; CHF, congestive heart failure; CVD, cardiovascular disease; CCI, Charlson Comorbidity Index; SGA, subjective global assessment; KDQOL-SF, kidney disease quality of life short form; RAS, renin-angiotensin system; CCB, calcium channel blocker; Ca, calcium; P, phosphorus; ESA, erythropoiesis- stimulating agent; TSAT, transferrin saturation.

When patients were divided into three groups according to baseline TSAT concentrations, age, sex, the proportion of patients on HD, the presence of DM and CV diseases, and KDQOL-SF scores were comparable among the three groups. There were also no differences in the proportions of patients on renin-angiotensin system (RAS) blockers, ESA, and supplementary iron. However, beta-blockers were more frequently prescribed in the lowest TSAT group compared to the other groups (P = 0.019) (Table 1). Even though only one third of patients (n = 319, 36.3%) were on iron therapy at the time of dialysis commencement, 166 patients with TSAT ≤20% (58.9%) were newly treated with iron agents after the initiation of dialysis. Therefore, 261 patients (92.6%) with initial TSAT ≤20% were on iron therapy at 3-month.

Within the laboratory and echocardiographic findings, blood urea nitrogen and creatinine levels were significantly lower in the lowest TSAT group compared to the highest TSAT group (P<0.001), whereas serum calcium, bicarbonate, and triglyceride concentrations were significantly higher (P<0.05). There were also significant gradual increases in serum iron and ferritin concentrations and a significant gradual decrease in TIBC levels across the TSAT groups (P<0.001). However, eGFR and Hb concentrations were not significantly different among the three groups. Meanwhile, hs-CRP and NT-proBNP levels were significantly higher in the lowest TSAT group compared to the other two groups (P<0.001 and P = 0.011, respectively). Echocardiographic parameters were comparable among the three groups (Table 2).

**Table 2:** Laboratory and echocardiographic findings of the patients.

Variables	Total	TSAT ≤20%	TSAT 20–40%	TSAT >40%	P
Baseline laboratory data					
WBC (/μL)	7239.1±2936.6	7637.2±3080.2	7055.1±2793.8	7039.6±2997.5	0.022
Hemoglobin (g/dL)	8.55±1.43	8.54±1.34	8.47±1.41	8.44±1.61	0.604
ALP (IU/L)	78.0 (59.0–109.0)	80.0 (60.0–109.0)	75.0 (57.0–105.0)	80.5 (59.0–115.3)	0.065
Ca (mg/dL)	8.22±1.08	8.32±0.96	8.22±1.05	8.06±1.30	0.048
P (mg/dL)	5.61±1.38	5.59±1.08	5.60±1.36	5.81±1.81	0.106
Ca x P product	45.4±15.1	45.4±13.7	45.3±14.9	46.1±17.7	0.239
Uric acid (mg/dL)	8.36±2.13	8.25±2.06	8.40±2.07	8.50±2.35	0.260
Glucose (mg/dL)	140.9±37.4	141.6±40.7	141.9±39.5	137.0±25.1	0.747
HbA1c (%)	6.12±0.81	6.12±0.90	6.16±0.75	6.02±0.81	0.570
Protein (g/dL)	6.05±0.80	6.08±0.76	6.03±0.82	6.06±0.82	0.702
Albumin (g/dL)	3.32±0.59	3.29±0.59	3.33±0.57	3.35±0.61	0.522
BUN (mg/dL)	82.7±26.8	76.6±22.7	82.3±25.7	94.4±33.4	<0.001
Creatinine (mg/dL)	8.52±3.52	7.97±2.58	8.42±3.32	9.73±4.98	<0.001
GFR-MDRD (mL/min/1.73m2)	7.12±3.13	7.40±3.42	7.18±3.14	6.51±2.54	0.080
GFR-EPI (mL/min/1.73m2)	7.29±2.94	7.47±2.76	7.39±3.16	6.72±2.60	0.111
Sodium (mEq/L)	137.2±4.9	137.0±4.6	137.3±5.0	137.4±5.0	0.763
Potassium(mEq/L)	4.67±0.95	4.61±0.95	4.66±0.93	4.79±1.01	0.115
Bicarbonate (mmol/L)	19.0±5.6	19.3±5.3	19.1±5.5	17.9±6.5	0.037
Intact-PTH (pg/mL)	203.9 (119.0–341.4)	198.7 (119.4–326.7)	210.0 (112.6–329.3)	209.9 (129.1–379.9)	0.413
Serum iron (μg/dL)	60.5±32.0	29.8±8.9	61.2±14.6	110.6±33.9	<0.001
TIBC (μg/dL)	217.7±44.0	227.7±51.9	216.5±39.5	204.2±37.2	<0.001
TSAT (%)	28.4±16.0	13.2±4.2	28.4±5.6	54.3±13.0	<0.001
Ferritin (ng/mL)	201.3 (103.5–363.4)	122.6 (64.3–274.1)	216.2 (121.6–369.8)	294.4 (190.3–479.9)	<0.001
Total cholesterol (mg/dL)	155.2±46.3	153.9±44.8	156.7±46.9	151.5±47.3	0.219
Triglyceride (mg/dL)	127.1±54.8	127.3±64.9	129.2±51.0	116.7±43.6	0.035
LDL-cholesterol (mg/dL)	89.3±32.0	87.8±29.3	92.6±34.7	83.6±28.6	0.044
HDL-cholesterol (mg/dL)	38.6±12.5	38.4±11.9	38.3±12.2	39.9±14.2	0.380
hs-CRP (mg/dL)	0.32 (0.08–1.46)	0.71 (0.15–2.09)	0.24 (0.06–0.99)	0.25 (0.07–1.20)	<0.001
Troponin-T (ng/mL)	0.056 (0.025–0.110)	0.057 (0.027–0.125)	0.059 (0.025–0.106)	0.046 (0.021–0.096)	0.409
NT-proBNP (pg/mL)	6505.4 (1576.7–25840.0)	11012.0 (1998.0–30000.0)	5969.5 (1364.0–21882.8)	4903.0 (1269.0–25151.5)	0.011
24-hr urine study					
Urea (mg/dL)	220.0 (142.0–327.4)	190.1 (136.8–293.5)	238.8 (152.5–347.3)	206.7 (127.8–325.1)	0.018
Creatinine (mg/dL)	54.1 (38.0–86.6)	51.5 (36.4–76.0)	55.9 (38.4–91.3)	56.2 (36.6–84.1)	0.119
Protein (mg/day)	1180.5 (418.1–2794.5)	1153.0 (426.2–3150.5)	1305.6 (393.0–2951.7)	1053.0 (383.0–2604.0)	0.723
Volume (mL/day)	970.0 (600.0–1400.0)	990.0 (525.0–1445.0)	985.0 (600.0–1450.0)	880.0 (600.0–1300.0)	0.367
Echocardiographic parameters					
LVPWT (cm)	1.20±0.29	1.21±0.29	1.19±0.29	1.20±0.27	0.737
IST (cm)	1.15±0.22	1.17±0.24	1.14±0.22	1.13±0.20	0.095
LVESD (cm)	3.51±0.72	3.58±0.75	3.48±0.71	3.43±0.69	0.062
LVEDD (cm)	5.16±0.61	5.20±0.61	5.15±0.60	5.12±0.63	0.329
LAD (cm)	4.22±0.71	4.27±0.71	4.22±0.71	4.13±0.69	0.131
LVMI (g/m2)	146.7±43.4	151.7±46.1	144.2±42.1	144.5±41.6	0.117
LVEF (%)	58.2±11.3	57.3±12.3	58.3±11.1	59.3±10.3	0.206

*Abbreviations*: WBC, white blood cell; ALP, alkaline phosphatase; Ca, calcium; P, phosphorus; HbA1C, hemoglobin A1C; BUN, blood urea nitrogen; GFR, glomerular filtration rate; MDRD, Modification of Diet in Renal Disease; EPI, Chronic Kidney Disease Epidemiology Collaboration Equation; PTH, parathyroid hormone;TIBC, total iron binding capacity; TSAT, transferrin saturation; LDL, low density lipoprotein; HDL, high density lipoprotein; hs-CRP, high-sensitivity C-reactive protein; NT-proBNP, N-terminal pro B-type natriuretic peptide; LVPWT, left ventricular posterior wall thickness; IST, inter-ventricularseptalthickness; LVESD, left ventricular end-systolic dimension; LVEDD, left ventricular end-diastolic dimension; LAD, left atrial dimension; LVMI, left ventricular mass index; LVEF, left ventricular ejection fraction.

### CV mortality, CV composite outcome, and all-cause mortality according to TSAT concentrations

During a mean follow-up duration of 19.3±11.8 months, 51 patients (5.8%) died. Among them, 29 patients (3.3%) died from CV causes and 12 (1.4%) from infection.

**Figure 1:**
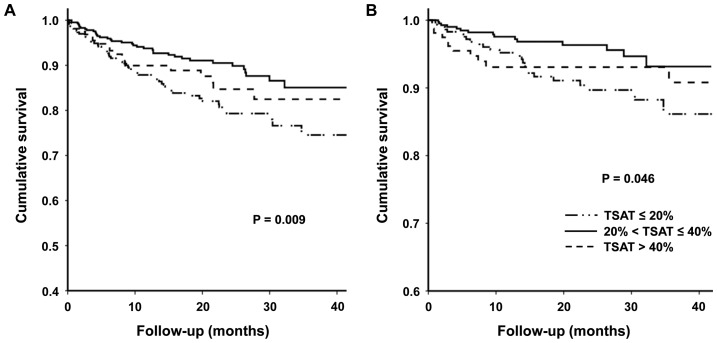
Kaplan-Meier curves for CV composite outcome (A) and all-cause mortality (B) according to baseline TSAT concentrations. The CV composites and all-cause mortality rates were significantly higher in patients with TSAT ≤20% compared to the other two groups. *Abbreviation:* CV, cardiovascular; TSAT, transferrin saturation.

Compared to the reference group (TSAT 20–40%), CV composite and all-cause mortality rates were significantly higher in patients with TSAT ≤20% (CV composite: 11.71 vs. 5.55 events per 100 patient-yr, P = 0.001; all-cause mortality: 5.38 vs. 2.31 events per 100 patient-yr, P = 0.016). In contrast, there was no significant difference in CV mortality rates among the three groups (Table 3). Kaplan-Meier analysis also showed that the CV composite and all-cause mortality rates were significantly higher in patients with TSAT ≤20% compared to the other two groups (P = 0.009 and P = 0.046, respectively) (Figure 1). However, there was no significant difference in the CV composite and all-cause mortality rates between the reference and TSAT >40% groups.

**Table 3:** Comparisons of clinical outcomes according to the TSAT concentrations.

	TSAT ≤20%		20% < TSAT ≤40%		TSAT >40%		
	N (%)	Rates (per 100 patient-yr)	N (%)	Rates (per 100 patient-yr)	N (%)	Rates (per 100 patient-yr)	P
CV mortality	12 (4.3%)	2.58	10 (2.3%)	1.44	7 (4.2%)	2.73	0.281
CV composite	51 (18.1%)	11.71	37 (8.6%)	5.55	19 (11.4%)	7.78	0.001
All-cause mortality	25 (8.9%)	5.38	16 (3.7%)	2.31	10 (6.0%)	3.90	0.016

*Composite: composite of death and hospitalization.

*Abbreviations*: TSAT, transferrin saturation; CV, cardiovascular.

Multivariate Cox-proportional hazard regression analysis revealed that patients in the lowest TSAT group had 1.62- and 2.19-fold higher risks for CV composite outcome and all-cause mortality, respectively, even after adjusting for demographic characteristics, laboratory findings, and echocardiographic parameters (P = 0.046 and P = 0.030, respectively) (Table 4).

**Table 4:** Hazard ratios and 95% confidence intervals for primary and secondary endpoints according to baseline TSAT concentrations (Cox-proportional hazard regression analysis).

	CV mortality			CV composite			All-cause mortality		
	HR	95% CI	P	HR	95% CI	P	HR	95% CI	P
Unadjusted									
TSAT ≤20%	1.798	0.777–4.161	0.171	1.918	1.256–2.929	0.003	2.117	1.130–3.965	0.019
20% < TSAT ≤40%	1	-	-	1	-	-	1	-	-
TSAT >40%	1.881	0.716–4.941	0.200	1.420	0.817–2.469	0.214	1.714	0.778–3.777	0.182
Adjusted for demographics									
TSAT ≤20%	1.651	0.710–3.841	0.245	1.729	1.123–2.661	0.013	2.278	1.190–4.363	0.013
20% < TSAT ≤40%	1	-	-	1	-	-	1	-	-
TSAT >40%	1.785	0.675–4.724	0.243	1.499	0.859–2.616	0.154	1.749	0.760–4.026	0.189
Adjusted for demographics and medications									
TSAT ≤20%	1.635	0.701–3.813	0.255	1.645	1.066–2.539	0.025	2.002	1.041–3.852	0.038
20% < TSAT ≤40%	1	-	-	1	-	-	1	-	-
TSAT >40%	1.797	0.679–4.755	0.238	1.488	0.851–2.603	0.163	1.814	0.790–4.165	0.160
Adjusted for demographics, medications, and laboratory parameters									
TSAT ≤20%	1.448	0.608–3.445	0.403	1.668	1.049–2.652	0.030	2.128	1.057–4.283	0.034
20% < TSAT ≤40%	1	-	-	1	-	-	1	-	-
TSAT >40%	1.587	0.583–4.315	0.366	1.323	0.751–2.331	0.332	1.432	0.613–3.343	0.407
Adjusted for demographics, medications, laboratory parameters, and echocardiographic findings									
TSAT ≤20%	1.383	0.555–3.448	0.486	1.616	1.008–2.607	0.046	2.193	1.081–4.450	0.030
20% < TSAT ≤40%	1	-	-	1	-	-	1	-	-
TSAT >40%	1.687	0.580–4.908	0.337	1.212	0.673–2.185	0.522	1.292	0.533–3.129	0.571

*Composite: composite of death and hospitalization.

*CV mortality was sequentially adjusted for demographics (age, diabetes, underlying cardiovascular disease, and subjective global assessment score), medications (aspirin), laboratory parameters (albumin, glucose, alkaline phosphatase, and log transformed high-sensitivity C-reactive protein), and echocardiographic findings (left ventricular mass index and left ventricular ejection fraction).

*CV composite was sequentially adjusted for demographics (age, systolic blood pressure, diabetes, and underlying cardiovascular disease), medications (aspirin, clopidogrel, vitamin D, and erythropoiesis-stimulating agents), laboratory parameters (calcium, glucose, potassium, log transformed ferritin, and log transformed high-sensitivity C-reactive protein), and echocardiographic findings (left ventricular mass index and left ventricular ejection fraction).

*All-cause mortality was sequentially adjusted for demographics (body mass index, Charlson Comorbidity Index, and subjective global assessment score), medications (aspirin, clopidogrel, and vitamin D), laboratory parameters (alkaline phosphatase, calcium, glucose, log transformed ferritin, and log transformed high-sensitivity C-reactive protein), and echocardiographic findings (left ventricular mass index and left ventricular ejection fraction).

*Abbreviations*: TSAT, transferrin saturation; CV, cardiovascular; HR, hazard ratio; CI, confidence interval.

In another analysis, we simply dichotomized patients into ‘low’ and ‘normal to high’ TSAT groups based on TSAT concentrations of 20%, and compared the clinical outcomes between the two groups. The ‘low’ TSAT group had significantly higher rates of CV composite (11.71 vs. 6.15 events per 100 patient-yr, P<0.001) and all-cause mortality (5.38 vs. 2.74 events per 100 patient-yr, P = 0.008) than patients with TSAT >20%, and the hazard ratios (HRs) were 1.53 and 2.04, respectively, on the multivariate Cox regression analysis (Table S1 and S2, and Figure S1).

### Relationships between TSAT and echocardiographic parameters

In binary logistic regression analysis for echocardiographic parameters, TSAT ≤20% was demonstrated to be significantly associated with LVH [odds ratio (OR)  = 1.46, P = 0.048]. The ORs of TSAT ≤20% for LVEF <60%, LVEDD ≥55 mm, LVESD ≥35 mm, and LAD ≥40 mm were also increased, but did not reach statistical significances (Table 5).

**Table 5:** Odds ratios and 95% confidence intervals for echocardiographic parameters according to baseline TSAT concentrations (Logistic regression analysis).

	Unadjusted			Adjusted		
	OR	95% CI	P	OR	95% CI	P
Left ventricular ejection fraction <60%						
TSAT ≤20%	1.435	1.068–1.926	0.016	1.313	0.910–1.895	0.146
20% < TSAT ≤40%	1	-	-	1	-	-
TSAT >40%	0.969	0.689–1.363	0.857	0.715	0.466–1.096	0.124
Left ventricular hypertrophy						
TSAT ≤20%	1.482	1.065–2.062	0.020	1.455	1.003–2.110	0.048
20% < TSAT ≤40%	1	-	-	1	-	-
TSAT >40%	0.838	0.574–1.224	0.361	0.931	0.604–1.435	0.747
Left ventricular end-diastolic dimension ≥55 mm						
TSAT ≤20%	1.363	0.981–1.892	0.065	1.258	0.849–1.862	0.252
20% < TSAT ≤40%	1	-	-	1	-	-
TSAT >40%	0.941	0.624–1.418	0.770	0.765	0.474–1.235	0.274
Left ventricular end-systolic dimension ≥35 mm						
TSAT ≤20%	1.346	0.995–1.821	0.054	1.341	0.935–1.924	0.111
20% < TSAT ≤40%	1	-	-	1	-	-
TSAT >40%	0.888	0.617–1.277	0.522	0.687	0.449–1.052	0.084
Left atrial dimension ≥40 mm						
TSAT ≤20%	1.456	1.051–2.015	0.024	1.145	0.777–1.689	0.494
20% < TSAT ≤40%	1	-	-	1	-	-
TSAT >40%	0.835	0.578–1.205	0.335	0.725	0.468–1.124	0.150

*Left ventricular ejection fraction was adjusted for sex, body mass index, heart rates, underlying diabetes and cardiovascular disease, hemoglobin, serum glucose, albumin, and creatinine levels, log transformed high-sensitivity C-reactive protein, and usage of diuretics, beta blockers, and vitamin D.

*Left ventricular hypertrophy was adjusted for sex, diastolic blood pressure, underlying cardiac disease, subjective global assessment score, hemoglobin, serum calcium, and albumin concentrations, and usage of renin-angiotensin system blockers and beta blockers.

*Left ventricular end-diastolic dimension was adjusted for sex, body mass index, underlying diabetes and cardiac disease, smoking status, hemoglobin, serum phosphorus and albumin levels, log transformed high-sensitivity C-reactive protein, and usage of renin-angiotensin system blockers and beta blockers.

*Left ventricular end-systolic dimension was adjusted for sex, diastolic blood pressure, underlying cardiovascular disease, smoking status, hemoglobin, serum phosphorus, albumin, and creatinine concentrations, log transformed high-sensitivity C-reactive protein, and usage of renin-angiotensin system blockers and beta blockers.

*Left atrial dimension was adjusted for age, sex, pulse pressure, underlying diabetes and cardiac disease, smoking status, hemoglobin, serum albumin levels, log transformed high-sensitivity C-reactive protein, and usage of diuretics, beta blockers, calcium channel blockers, and aspirin.

*Abbreviations*: TSAT, transferrin saturation; OR, odds ratio; CI, confidence interval.

Based on these results, we performed an additional multivariate linear regression analysis for LVMI. Along with diastolic blood pressure, underlying CV disease, SGA score, smoking status, serum Hb and albumin concentrations, and usage of RAS blockers, calcium-based phosphate binders, and iron agents, TSAT ≤20% was a significant independent determinant of LVMI (R = 7.151, P = 0.044) (Table S3).

### Relationships between TSAT and serum inflammatory and cardiac biomarkers

Binary logistic regression analysis revealed that there were significant independent associations of TSAT ≤20% with hs-CRP ≥3 mg/dL (OR = 2.09, P = 0.003), NT-proBNP ≥10,000 pg/mL (OR  = 2.04, P = 0.011), and cTnT ≥0.1 ng/mL (OR  = 2.02, P = 0.023) (Table 6).

**Table 6:** Odds ratios and 95% confidence intervals for serum inflammatory and cardiac biomarkers according to baseline TSAT concentrations (Logistic regression analysis).

	Unadjusted			Adjusted		
	OR	95% CI	P	OR	95% CI	P
hs-CRP ≥3 mg/dL						
TSAT ≤20%	1.772	1.162–2.702	0.008	2.087	1.292–3.372	0.003
20% < TSAT ≤40%	1	-	-	1	-	-
TSAT >40%	0.980	0.558–1.722	0.945	0.817	0.443–1.506	0.517
NT-proBNP ≥10000 pg/mL						
TSAT ≤20%	1.630	1.124–2.363	0.010	2.039	1.181–3.521	0.011
20% < TSAT ≤40%	1	-	-	1	-	-
TSAT >40%	0.640	0.400–1.023	0.062	0.328	0.171–0.629	0.001
Troponin-T ≥0.1 ng/mL						
TSAT ≤20%	1.498	1.004–2.235	0.048	2.019	1.104–3.691	0.023
20% < TSAT ≤40%	1	-	-	1	-	-
TSAT >40%	0.876	0.518–1.484	0.623	0.989	0.484–2.024	0.976

*hs-CRP was adjusted for Charlson Comorbidity Index, subjective global assessment score, smoking status, hemoglobin, serum calcium, glucose, albumin, and sodium concentrations, and log transformed ferritin.

*NT-proBNP was adjusted for diastolic blood pressure, underlying diabetes and cardiac disease, hemoglobin, serum glucose, albumin, creatinine, and sodium levels, log transformed high-sensitivity C-reactive protein, log transformed ferritin, log transformed troponin-T, and usage of renin-angiotensin system blockers, diuretics, and beta blockers.

*Troponin-T was adjusted for sex, systolic blood pressure, underlying diabetes and cardiac disease, subjective global assessment score, hemoglobin, serum calcium, glucose, and albumin concentrations, log transformed high-sensitivity C-reactive protein, log transformed ferritin, log transformed 24-hr urine volume, left atrial dimension, left ventricular mass index, left ventricular ejection fraction, and usage of diuretics and nitrate.

*Abbreviations*: hs-CRP, high-sensitivity C-reactive protein; NT-proBNP, N-terminal pro B-type natriuretic peptide; TSAT, transferrin saturation; OR, odds ratio; CI, confidence interval.

In an additional multivariate linear regression analysis, TSAT ≤20% was found to be a significant determinant of natural log values (Ln) of hs-CRP levels (R = 0.304, P<0.001), but not of Ln NT-proBNP (R = 0.229, P = 0.134) and Ln cTnT concentrations (R = 0.092, P = 0.406) (Table S4).

## Discussion

TSAT is one of the available markers reflecting the adequacy of iron for erythropoiesis and is closely correlated with Hb levels [9], [11]. Even though a number of studies have shown that anemia is an independent risk factor for CV events and mortality in ESRD patients, as well as in the general population [3], [4], [7], [25], the impact of TSAT on clinical outcome has never been explored in dialysis patients. In the current study on incident dialysis patients with anemia, we demonstrate for the first time that the lowest TSAT group has significantly higher risk for CV composite outcome and all-cause mortality, irrespective of Hb concentrations. In addition, TSAT ≤20% was significantly associated with LVMI and hs-CRP levels. These findings suggest that the adverse clinical outcomes in patients with ‘low’ TSAT are partly attributed to LVH and inflammation.

Iron deficiency is the most frequent cause of anemia in the general population. In ESRD patients, a decrease in the production of erythropoietin is the main factor contributing to anemia, but iron deficiency anemia (IDA) is also prevalent [8]. Even though anemia has been demonstrated to be an independent risk factor for renal function deterioration and CV morbidity/mortality in patients with CKD [4], [7], [25], the results of some previous studies on the impact of anemia correction on the clinical outcome of CKD/ESRD patients have not been promising, except for some improvement in quality of life [26]–[28]. Similarly, anemia is common in patients with CHF, most of which are attributed to ‘anemia of chronic disease’ or iron deficiency [5], [29], [30]. Numerous previous studies demonstrated that anemia was associated with impaired functional capacity and poor quality of life in CHF patients, which were ameliorated by elimination of the anemia [31], [32]. However, a body of evidence indicating that anemia correction has a beneficial effect on CV mortality in these patients is lacking. Based on these findings, it is still debatable whether adverse clinical outcomes in anemic patients are attributed to anemia per se or other factors contributing to anemia.

Iron is essential for normal cell physiology. In addition to its critical role in erythropoiesis, iron is involved in the process of oxygen transport and storage, oxidative metabolism including ATP (adenosine triphosphate) production at the mitochondrial electron transport chain in the form of cytochrome (a, b, c) and iron-sulfur-containing dehydrogenases, and the synthesis and/or degradation of RNA and DNA. Therefore, iron deficiency can lead to not only anemia but also various cellular dysfunctions, especially in high energy-requiring cells such as cardiomyocytes and renal cells. In the clinical field, reduced oxidative capacity caused by cytochrome dysfunction can manifest as fatigue, impaired prolonged exercise, malnutrition, and cardiac dysfunction [12], [33], [34]. A previous study by Dong et al. [13] showed that heart weight and size were significantly increased, and the left ventricular dimension and chamber volume were significantly enhanced, in rats fed an iron-deficient diet for 12 weeks. Mitochondrial swelling and abnormal sarcomere structure were also observed in ventricular tissues of the iron-deficient rats. These findings inferred that iron deficiency per se might induce cardiac dysfunction and morphological aberration. Moreover, another recent study demonstrated that patients with advanced CHF displayed significantly lower serum iron concentrations and TSAT compared to healthy controls. This study also showed that myocardial iron content and myocardial mRNA expression of the type 1 transferrin receptor, a key molecule in cellular iron transport, were significantly reduced in CHF versus non-CHF samples, suggesting a linkage among the presence of iron depletion in the failing heart, anemia, and adverse prognosis in CHF [35]. However, since hematocrit or Hb levels were significantly lower in the iron-deficiency groups in those two studies, it was difficult to conclude whether iron deficiency had a direct unfavorable impact on the heart.

Recently, Jankowska et al. [15] found that iron deficiency, defined as ferritin <100 μg/L or ferritin 100-300 μg/L with TSAT <20%, rather than anemia, was an independent risk factor for poor clinical outcome in 546 patients with stable systolic CHF. Patients with iron deficiency had significantly lower survival rates, either when death and heart transplantation or death alone were considered events in both univariate and multivariate Cox regression models, whereas anemia was a significant predictor of adverse clinical outcome only in univariate analysis. Based on these findings, they suggested that iron deficiency had a direct deleterious effect on cardiomyocytes. The results of the present study also showed that clinical outcome was significantly worse in the lowest TSAT group, despite comparable Hb concentrations, and that TSAT ≤20% was a significant independent risk factor for CV composite outcome and all-cause mortality. Taken together, we surmise that the association between ‘low’ TSAT and adverse clinical outcome in incident dialysis patients is a consequence of iron deficiency rather than anemia in the heart, which may be partly attributed to mitochondrial cytochrome dysfunction.

In a similar context, several clinical trials have shown the beneficial effect of iron replacement therapy in CHF patients with anemia. The first randomized trial was performed by Toblli et al. [31] to evaluate the effect of intravenous iron therapy in anemic patients with CHF and CKD, and demonstrated that iron supplements substantially reduced NT-proBNP and inflammatory status in these patients, along with an improvement in LVEF, New York Heart Association (NYHA) functional class, exercise capacity, and quality of life. However, they found that Hb levels were also significantly increased in the treatment group compared to the placebo group. Recently, two prospective randomized controlled trials showed that the correction of iron deficiency with intravenous iron conferred symptomatic benefits in patients with CHF [36], [37]. Compared to the control group, patient global assessment, exercise capacity, and NYHA functional class were significantly improved in the iron-treated group regardless of the presence of anemia, suggesting that iron deficiency per se was detrimental to the heart. Considering the results of our study, further studies on the use of intravenous iron in incident ESRD patients with ‘low’ TSAT will be necessary to clarify whether iron therapy can improve the clinical outcome of these patients, as in CHF patients.

Meanwhile, an excess of iron can be toxic because it has the ability to accept and donate electrons by exchanging between ferrous and ferric forms. During this exchange, active free iron undergoes “Fenton and Haber-Weiss reaction”, causing oxidative stress and organic biomolecule oxidation by releasing hydroxyl radicals and other reactive oxygen species (ROS) [38], [39]. Furthermore, in the vasculature, ROS produced at the endothelium by excessive iron is known to promote the thrombotic complications [33]. Even though ferritin concentration is usually considered a marker of iron store and has been demonstrated to be an independent predictor of clinical outcomes in not only the general population but also specific patients groups, several previous studies have found that TSAT, an indicator of a predisposition for iron overload, is also a significant prognostic factor in the general population. A cohort study using data from the First Health and Nutrition Examination Survey I (NHANES I) merged with the NHANES I Epidemiologic Follow Up Study showed that all-cause mortality was significantly increased in patients with a serum TSAT of more than 55% compared with those with saturations below this cutoff [HR  = 1.60, 95% confidence interval (CI)  = 1.17–2.21], on a Cox proportional hazard regression analysis [40]. In addition, Ellervik et al. [41] examined mortality according to baseline TSAT in two Danish population-based follow-up studies and demonstrated that the cumulative survival was significantly reduced in individuals with TSAT ≥50% vs. <50% (P<0.0001). In that study, there was a stepwise increase in all-cause mortality, with the first significant increased risk conferred by TSAT ≥40%. Based on these findings, we tried to elucidate the impact of iron deficiency as well as a proxy of iron overload, and thus categorized patients into 3 groups based on TSAT; ≤20% (low), 20–40% (normal), and >40% (high). Even though HRs for clinical outcomes; CV mortality, CV composite, and all-cause mortality; were slightly increased in patients with TSAT >40%, there were no statistical significances. In another analysis conducted after dichotomizing the study subjects into just ‘low’ and ‘normal to high’ TSAT groups, patients with TSAT ≤20% showed significantly higher rates of CV composite outcome and all-cause mortality, and the HRs for each outcome were 1.53 and 2.04, respectively, which were comparable with those of the original analysis. These data indirectly implicate the lack of statistical difference between the reference group and subjects with TSAT >40%, and further clarify our finding of low TSAT (≤20%) as a risk factor for adverse clinical outcomes.

LVH is a well-known powerful independent predictor of CV morbidity and mortality in patients with ESRD. Furthermore, a change in LVH has been identified as a strong prognostic factor in these patients. A previous prospective study on prevalent HD patients revealed that the rates of LVMI increase were significantly higher in patients with incident CV events than in those without such events and that cardiovascular event-free survival in patients with changes in LVMI below the 25th percentile was significantly higher than in those with changes above the 75th percentile [42]. Similarly, in a cohort study of 153 incident ESRD patients receiving HD, a reduction in LV mass during a mean follow-up duration of 54 months resulted in significant decreases in all-cause and CV mortality. In that study, LV mass reduction was also independently associated with improved patient survival, even after adjustment for confounding variables [43]. On the other hand, uremia-related nontraditional risk factors, including inflammation and oxidative stress, were implicated in the pathogenesis of CV disease in dialysis patients [44]. Since accumulating evidence indicates that inflammation is an integral part of the development and progression of atherosclerosis, it has been proposed that hs-CRP concentrations are closely linked to the presence of CV disease. In addition, numerous previous studies have found that serum hs-CRP levels are predictive of CV mortality, as well as future CV events, in not only the general population but also ESRD patients [45], [46]. In this study, LVMI and serum hs-CRP concentrations were higher in the lowest TSAT group compared to the other two groups. Moreover, TSAT ≤20% was found to be a significant determinant of LVMI and Ln hs-CRP concentrations in multivariate linear regression analysis. Taken together, the impact of ‘low’ TSAT on clinical outcome in our patients seems to be partly mediated by LVH and inflammation.

Several shortcomings of the current study should be discussed. First, since the study subjects were all Korean incident ESRD patients, the association of TSAT with mortality and composite outcome may not be generalizable to other populations. Second, because only the baseline laboratory and echocardiographic measurements were used for the analysis, it was difficult to demonstrate the impact of the changes of TSAT on patients' clinical outcomes. Third, even though CV disease was the leading cause of death in our subjects, CV mortality was comparable among the three groups in spite of a significant difference in all-cause mortality, which may be attributed to ‘low’ CV mortality rates in the current study compared to those in previous studies on Western ESRD patients. We propose that the difference is mainly attributed to disparate ethnicities as the mortality rates of our patients were not significantly different from those of Japanese dialysis patients [47]. Fourth, we stratified the patients based on TSAT only, without considering ferritin levels, indicating that patients with TSAT ≤20% may not be purely iron deficient. Since ferritin is an acute-phase reactant, high ferritin concentrations with low TSAT may imply a condition of iron sequestration, which is a characteristic of ‘anemia of chronic disease’. In the present study, in fact, there was a significant correlation between serum ferritin levels and Ln hs-CRP concentrations (r = 0.368, P<0.001), whereas Hb levels did not correlate with serum ferritin concentrations (r = 0.002, P = 0.954). Fifth, TSAT is also known to be influenced by nutritional status. TIBC may be decreased due to reduced transferrin synthesis in the setting of malnutrition and chronic disease, resulting in high TSAT, disproportionate to the iron content [11]. However, serum albumin levels were not significantly different among the three groups, suggesting that TSAT was not largely affected by nutritional status in our patients. Sixth, data for transfusion were not available. So, we excluded patients who had a history of acute/recent bleeding in the analysis. Lastly, about one-third of patients were already taking iron or ESA agents at the time of inclusion. Nevertheless, the proportions of patients taking those medications were not significantly different among the three groups, and multivariate analysis was conducted after adjusting for these factors. Despite these limitations, to our knowledge, the current study is the first study to investigate the impact of TSAT at the time of dialysis initiation on clinical outcome in a large and single-ethnicity incident dialysis patient cohort. Further studies will be needed to elucidate whether serial monitoring, rather than a single measurement of TSAT, is helpful in identifying ESRD patients at a high risk of all-cause and CV morbidity and mortality and whether iron supplements are beneficial to clinical outcome in ‘low’ TSAT patients.

In conclusion, ‘low’ TSAT was a significant independent risk factor for CV composite outcome and all-cause mortality in incident dialysis patients with anemia. Furthermore, TSAT ≤20% was significantly associated with LVMI and hs-CRP concentrations. These findings suggest that the adverse clinical outcomes in patients with ‘low’ TSAT are partly attributed to LVH and inflammation.

## Supporting Information

Figure S1 **Kaplan-Meier curves for CV composite outcome (A) and all-cause mortality (B) according to baseline TSAT concentrations.** The CV composites and all-cause mortality rates were significantly higher in patients with TSAT ≤20% compared to patients with TSAT >20%. *Abbreviation:* CV, cardiovascular; TSAT, transferrin saturation.
(TIF)

Table S1 **Comparisons of clinical outcomes between patients with TSAT ≤20% and TSAT >20%.**
(DOC)

Table S2 **Hazard ratios and 95% confidence intervals for primary and secondary endpoints according to baseline TSAT concentrations (Cox-proportional hazard regression analysis).**
(DOC)

Table S3 **Multivariate linear regression analysis for left ventricular mass index.**
(DOC)

Table S4 **Multivariate linear regression analyses for inflammatory and cardiac biomarkers.**
(DOC)
