# Supplementary figures and images for: The Relationship of Initial Transferrin Saturation to Cardiovascular Parameters and Outcomes in Patients Initiating Dialysis
Source: PLoS One. 2014 Feb 5;9(2):e87231. doi: 10.1371/journal.pone.0087231 (PMC3914817; doi:10.1371/journal.pone.0087231)

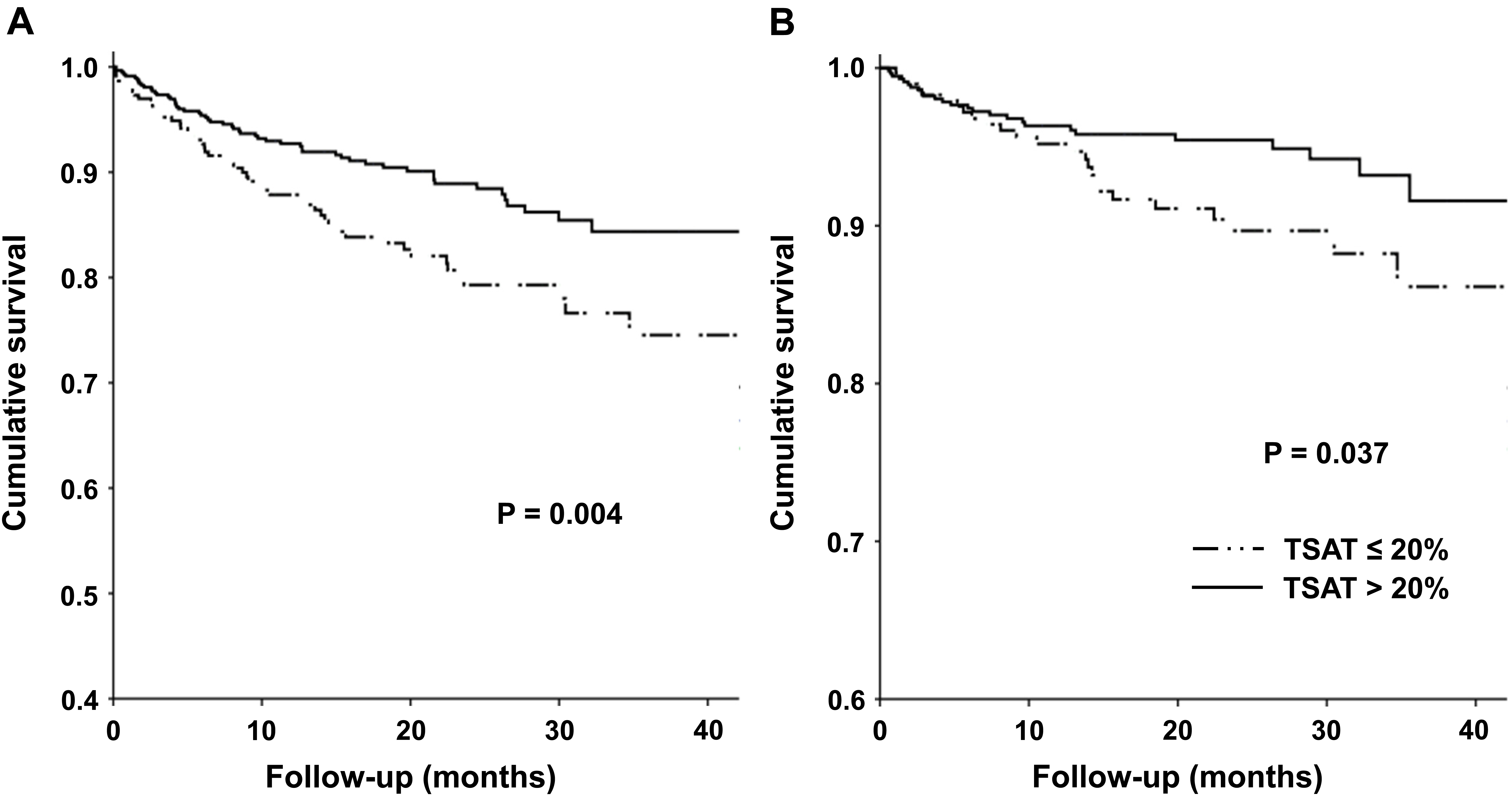

Supplement: Figure S1 — Kaplan-Meier curves for CV composite outcome (A) and all-cause mortality (B) according to baseline TSAT concentrations. The CV composites and all-cause mortality rates were significantly higher in patients with TSAT ≤20% compared to patients with TSAT >20%. Abbreviation: CV, cardiovascular; TSAT, transferrin saturation. (TIF) [file pone.0087231.s001.tif]
