# Supplementary material for: The Relationship of Initial Transferrin Saturation to Cardiovascular Parameters and Outcomes in Patients Initiating Dialysis
Source: PLoS One. 2014 Feb 5;9(2):e87231. doi: 10.1371/journal.pone.0087231 (PMC3914817; doi:10.1371/journal.pone.0087231)
Supplement: Table S1 — Comparisons of clinical outcomes between patients with TSAT ≤20% and TSAT >20%. (DOC) [file pone.0087231.s002.doc]

**Table S1.** Comparisons of clinical outcomes between patients with TSAT ≤ 20% and TSAT > 20%

|  | TSAT ≤ 20% | | TSAT > 20% | |  |
| --- | --- | --- | --- | --- | --- |
|  | N (%) | Rates (per 100 patient-yr) | N (%) | Rates (per 100 patient-yr) | P |
| CV mortality | 12 (4.3%) | 2.58 | 17 (2.8%) | 1.79 | 0.275 |
| CV composite | 51 (18.1%) | 11.71 | 56 (9.4%) | 6.15 | <0.001 |
| All-cause mortality | 25 (8.9%) | 5.38 | 26 (4.4%) | 2.74 | 0.008 |

*Composite: composite of death and hospitalization

*Abbreviations*: TSAT, transferrin saturation; CV, cardiovascular
