# Supplementary material for: The Relationship of Initial Transferrin Saturation to Cardiovascular Parameters and Outcomes in Patients Initiating Dialysis
Source: PLoS One. 2014 Feb 5;9(2):e87231. doi: 10.1371/journal.pone.0087231 (PMC3914817; doi:10.1371/journal.pone.0087231)
Supplement: Table S2 — Hazard ratios and 95% confidence intervals for primary and secondary endpoints according to baseline TSAT concentrations (Cox-proportional hazard regression analysis). (DOC) [file pone.0087231.s003.doc]

**Table S2.** Hazard ratios and 95% confidence intervals for primary and secondary endpoints according to baseline TSAT concentrations (Cox-proportional hazard regression analysis)

|  | CV mortality | | | CV composite | | | All-cause mortality | | |
| --- | --- | --- | --- | --- | --- | --- | --- | --- | --- |
|  | HR | 95% CI | P | HR | 95% CI | P | HR | 95% CI | P |
| Model 1 | 1.505 | 0.719-3.151 | 0.279 | 1.726 | 1.181-2.522 | 0.005 | 1.778 | 1.026-3.078 | 0.040 |
| Model 2 | 1.380 | 0.657-2.902 | 0.395 | 1.537 | 1.044-2.261 | 0.029 | 1.906 | 1.080-3.364 | 0.026 |
| Model 3 | 1.367 | 0.648-2.885 | 0.412 | 1.476 | 1.001-2.176 | 0.049 | 1.663 | 0.936-2.954 | 0.083 |
| Model 4 | 1.255 | 0.584-2.694 | 0.560 | 1.553 | 1.015-2.376 | 0.042 | 1.902 | 1.010-3.584 | 0.047 |
| Model 5 | 1.184 | 0.527-2.658 | 0.683 | 1.534 | 1.003-2.350 | 0.047 | 2.037 | 1.064-3.902 | 0.032 |

*Composite: composite of death and hospitalization

*Model 1: unadjusted (TSAT ≤ 20% versus TSAT > 20%)

Model 2: adjusted for demographics

Model 3: adjusted for Model 2 plus medications

Model 4: adjusted for Model 3 plus laboratory parameters

Model 5: adjusted for Model 4 plus echocardiographic findings

*CV mortality was sequentially adjusted for demographics (age, diabetes, underlying cardiovascular disease, and subjective global assessment score), medications (aspirin), laboratory parameters (albumin, glucose, alkaline phosphatase, and log transformed high-sensitivity C-reactive protein), and echocardiographic findings (left ventricular mass index and left ventricular ejection fraction).

*CV composite was sequentially adjusted for demographics (age, systolic blood pressure, diabetes, and underlying cardiovascular disease), medications (aspirin, clopidogrel, vitamin D, and erythropoiesis-stimulating agents), laboratory parameters (calcium, glucose, potassium, log transformed ferritin, and log transformed high-sensitivity C-reactive protein), and echocardiographic findings (left ventricular mass index and left ventricular ejection fraction).

*All-cause mortality was sequentially adjusted for demographics (body mass index, Charlson Comorbidity Index, and subjective global assessment score), medications (aspirin, clopidogrel, and vitamin D), laboratory parameters (alkaline phosphatase, calcium, glucose, log transformed ferritin, and log transformed high-sensitivity C-reactive protein), and echocardiographic findings (left ventricular mass index and left ventricular ejection fraction).

*Abbreviations*: TSAT, transferrin saturation; CV, cardiovascular; HR, hazard ratio; CI, confidence interval
