# Supplementary material for: The Relationship of Initial Transferrin Saturation to Cardiovascular Parameters and Outcomes in Patients Initiating Dialysis
Source: PLoS One. 2014 Feb 5;9(2):e87231. doi: 10.1371/journal.pone.0087231 (PMC3914817; doi:10.1371/journal.pone.0087231)
Supplement: Table S3 — Multivariate linear regression analysis for left ventricular mass index. (DOC) [file pone.0087231.s004.doc]

**Table S3.** Multivariate linear regression analysis for left ventricular mass index

| Variables | Regression coefficient | P |
| --- | --- | --- |
| Diastolic blood pressure (mmHg) | 0.289 | 0.006 |
| Cardiovascular disease | 16.773 | <0.001 |
| SGA > 1 (vs. SGA ≤ 1) | 17.028 | <0.001 |
| Current smoker (vs. ex-/non-smoker) | 10.291 | 0.036 |
| Hemoglobin (g/dL) | -2.650 | 0.021 |
| Albumin (g/dL) | -5.436 | 0.048 |
| Potassium (mEq/L) | 2.122 | 0.217 |
| TSAT (%) |  |  |
| TSAT ≤ 20% (vs. 20% < TSAT ≤ 40%) | 7.151 | 0.044 |
| TSAT > 40% (vs. 20% < TSAT ≤ 40%) | 0.576 | 0.892 |
| Renin-angiotensin system blockers | 16.472 | <0.001 |
| Beta blockers | 3.929 | 0.226 |
| Clopidogrel | 6.226 | 0.296 |
| Calcium-based phosphate binders | 7.449 | 0.023 |
| Erythropoietin stimulating agents | -0.996 | 0.778 |
| Iron agents | -8.783 | 0.010 |

*Abbreviations*: SGA, subjective global assessment; TSAT, transferrin saturation
