# Supplementary material for: The Relationship of Initial Transferrin Saturation to Cardiovascular Parameters and Outcomes in Patients Initiating Dialysis
Source: PLoS One. 2014 Feb 5;9(2):e87231. doi: 10.1371/journal.pone.0087231 (PMC3914817; doi:10.1371/journal.pone.0087231)
Supplement: Table S4 — Multivariate linear regression analyses for inflammatory and cardiac biomarkers. (DOC) [file pone.0087231.s005.doc]

**Table S4.** Multivariate linear regression analyses for inflammatory and cardiac biomarkers

(a) Log transformed high-sensitivity C-reactive protein (mg/dL)

| Variables | Regression coefficient | P |
| --- | --- | --- |
| Age (years) | 0.007 | 0.005 |
| Cardiovascular disease | 0.154 | 0.058 |
| SGA > 1 (vs. SGA ≤ 1) | 0.028 | 0.704 |
| Current smoker (vs. ex-/non-smoker) | 0.001 | 0.992 |
| Hemoglobin (g/dL) | -0.037 | 0.139 |
| Alkaline phosphatase (IU/L) | 0.078 | 0.233 |
| Calcium x Phosphorus product | 0.003 | 0.274 |
| Albumin (g/dL) | -0.153 | 0.012 |
| Sodium (mEq/L) | -0.023 | <0.001 |
| HDL-cholesterol (mg/dL) | -0.009 | 0.001 |
| Ln 24-hr urine volume | -0.088 | 0.065 |
| Ln ferritin | 0.310 | <0.001 |
| Ln troponin-T | 0.060 | 0.074 |
| TSAT (%) |  |  |
| TSAT ≤ 20% (vs. 20% < TSAT ≤ 40%) | 0.304 | <0.001 |
| TSAT > 40% (vs. 20% < TSAT ≤ 40%) | -0.145 | 0.114 |
| Left atrial dimension (cm) | 0.014 | 0.788 |
| Left ventricular ejection fraction (%) | -0.003 | 0.305 |
| Aspirin | 0.070 | 0.417 |

*Abbreviations*: SGA, subjective global assessment; HDL, high density lipoprotein; TSAT, transferrin saturation

(b) Log transformed N-terminal pro B-type natriuretic peptide (pg/mL)

| Variables | Regression coefficient | P |
| --- | --- | --- |
| Body mass index (kg/m^2^) | -0.076 | <0.001 |
| Systolic blood pressure (mmHg) | 0.005 | 0.063 |
| Chronic lung disease | 0.462 | 0.069 |
| Cardiovascular disease | 0.036 | 0.809 |
| Hemoglobin (g/dL) | -0.169 | 0.001 |
| Phosphorous (mg/dL) | 0.026 | 0.546 |
| Albumin (g/dL) | -0.475 | <0.001 |
| Creatinine (mg/dL) | 0.052 | 0.012 |
| Sodium (mEq/L) | -0.009 | 0.505 |
| Ln hs-CRP | 0.262 | 0.005 |
| Ln ferritin | 0.306 | 0.070 |
| TSAT (%) |  |  |
| TSAT ≤ 20% (vs. 20% < TSAT ≤ 40%) | 0.229 | 0.134 |
| TSAT > 40% (vs. 20% < TSAT ≤ 40%) | -0.172 | 0.344 |
| Left atrial dimension (cm) | 0.512 | <0.001 |
| Left ventricular mass index (g/m^2^) | 0.004 | 0.001 |
| Left ventricular ejection fraction (%) | -0.039 | <0.001 |
| Diuretics | 0.386 | 0.005 |
| Beta blockers | 0.279 | 0.042 |

*Abbreviations*: hs-CRP, high-sensitivity C-reactive protein; TSAT, transferrin saturation

(c) Log transformed cardiac Troponin-T (ng/mL)

| Variables | Regression coefficient | P |
| --- | --- | --- |
| Age (years) | 0.004 | 0.260 |
| Sex (Female vs. Male) | -0.214 | 0.034 |
| Pulse pressure (mmHg) | 0.007 | 0.009 |
| Diabetes | 0.426 | <0.001 |
| Cardiovascular disease | 0.065 | 0.581 |
| SGA > 1 (vs. SGA ≤ 1) | 0.160 | 0.146 |
| Hemoglobin (g/dL) | -0.076 | 0.034 |
| Calcium x Phosphorus product | 0.001 | 0.839 |
| Glucose (mg/dL) | 0.134 | 0.227 |
| Albumin (g/dL) | -0.187 | 0.027 |
| Sodium (mEq/L) | -0.018 | 0.063 |
| Ln hs-CRP | 0.133 | 0.053 |
| Ln ferritin | 0.344 | 0.005 |
| Ln 24-hr urine volume | -0.163 | 0.017 |
| TSAT (%) |  |  |
| TSAT ≤ 20% (vs. 20% < TSAT ≤ 40%) | 0.092 | 0.406 |
| TSAT > 40% (vs. 20% < TSAT ≤ 40%) | 0.109 | 0.410 |
| Left atrial dimension (cm) | 0.232 | 0.002 |
| Left ventricular mass index (g/m^2^) | 0.001 | 0.170 |
| Left ventricular ejection fraction (%) | -0.012 | 0.006 |
| Diuretics | 0.259 | 0.010 |
| Beta blockers | 0.127 | 0.195 |
| Nitrate | 0.222 | 0.347 |
| Aspirin | 0.075 | 0.546 |

*Abbreviations*: SGA, subjective global assessment; hs-CRP, high-sensitivity C-reactive protein; TSAT, transferrin saturation
